# GLORIF1, a global river flow dataset created by integrating process-based modelling and machine learning

**DOI:** 10.1038/s41597-026-07658-6

**Published:** 2026-07-04

**Authors:** Sümeyye Büşra Işık, Oriol Pomarol Moya, Michele Magni, Madlene Nussbaum, Derek Karssenberg, Edwin H. Sutanudjaja

**Affiliations:** 1 Department of Physical Geography, Faculty of Geosciences, Utrecht University, Utrecht, The Netherlands; 2 Istanbul Technical University, Istanbul, Türkiye; 3 State Hydraulic Works, Ankara, Türkiye

**Keywords:** Hydrology, Environmental sciences

## Abstract

Accurate streamflow data are essential for managing water resources and enhancing climate resilience worldwide. Yet, existing global streamflow datasets often lack accuracy or rely on sparse observations. To address these challenges, we present GLORIF1 (GLObal RIver Flow version 1), a comprehensive monthly river discharge dataset spanning 1979-2019. GLORIF1 is generated using a hybrid modeling approach that integrates the PCR-GLOBWB (PCRaster GLOBal Water Balance) hydrological model with a Random Forest (RF) algorithm. The dataset is trained on observation data from the Global River Discharge (GRDC) and validated with the independent data from the GSIM (Global Streamflow Indices and Metadata Archive) and RSEG (Remote Sensing-based Extension for the GRDC). With its global coverage and public accessibility, GLORIF1 can be used to support more accurate hydrological and water-related studies, making it a valuable resource for global hydrology studies.

## Background & Summary

The hydrological cycle is an essential component of the Earth’s systems, supporting ecosystems, regulating climate, and sustaining life on Earth. A fundamental element of this cycle, streamflow plays a crucial role in shaping landscapes, providing habitat for aquatic and riparian ecosystems, and supplying water for human use and agriculture^1,2^. Streamflow is also integral to the Earth’s climate system, influencing weather patterns and acting as a key variable in the global water and energy cycles^3^. Hence, streamflow or river discharge datasets are crucial for effective water resource management, ecosystem conservation, and climate resilience^4^.

Available streamflow datasets are either generated with process-based models or derived from observational data. Process-based models simulate the hydrological cycle using mathematical representations of physical processes, providing complete spatial and temporal coverage, but often pose challenges for mimicking observation data^5–7^. Conversely, observational data, which are considered more accurate, are limited by their spatial and temporal coverages, as they are being confined to locations where measurements are available and periods when they are recorded^8,9^. To overcome such issues, observational datasets may rely on interpolation to fill their spatio-temporal gaps, but this can produce large errors in data-scarce regions^10^. Some datasets attempt to integrate process-based models and observational data but are typically constrained to local or national scales^11–13^. Here, we present an improved global extent streamflow dataset that overcomes abovementioned challenges associated with current existing datasets.

The lack of datasets that effectively combine observational data with process-based models has been a significant limitation, as it prevents the creation of comprehensive global datasets that accurately reflect both the physical processes and the observed reality. A hybrid modeling approach offers a solution to this problem by integrating the strengths of observational data and process-based models through machine learning techniques^14^. This integration improves the accuracy and reliability of predictions by leveraging the capacity of machine learning algorithms to identify complex patterns and relationships in the data that may be overlooked by traditional process-based modeling.

Earlier studies by Shen *et al*.^15^ and Magni *et al*.^16^ developed a hybrid model that combines PCR-GLOBWB (PCRaster GLOBal Water Balance)^7^, a process-based hydrological model, with a machine learning algorithm, in particular Random Forest (RF). The framework was first tested at three stations in the Rhine catchment, using state variables and meteorological data from PCR-GLOBWB as predictors^15^. This was then extended to the global extent, adding catchment attributes as predictors to model discharge at about 2,000 stations worldwide^16^. These initial applications of RF integrated with PCR-GLOBWB river discharge simulation at specific Global Runoff Data Centre (GRDC)^8^ station locations have demonstrated improved accuracy in streamflow predictions.

**Fig. 1:**
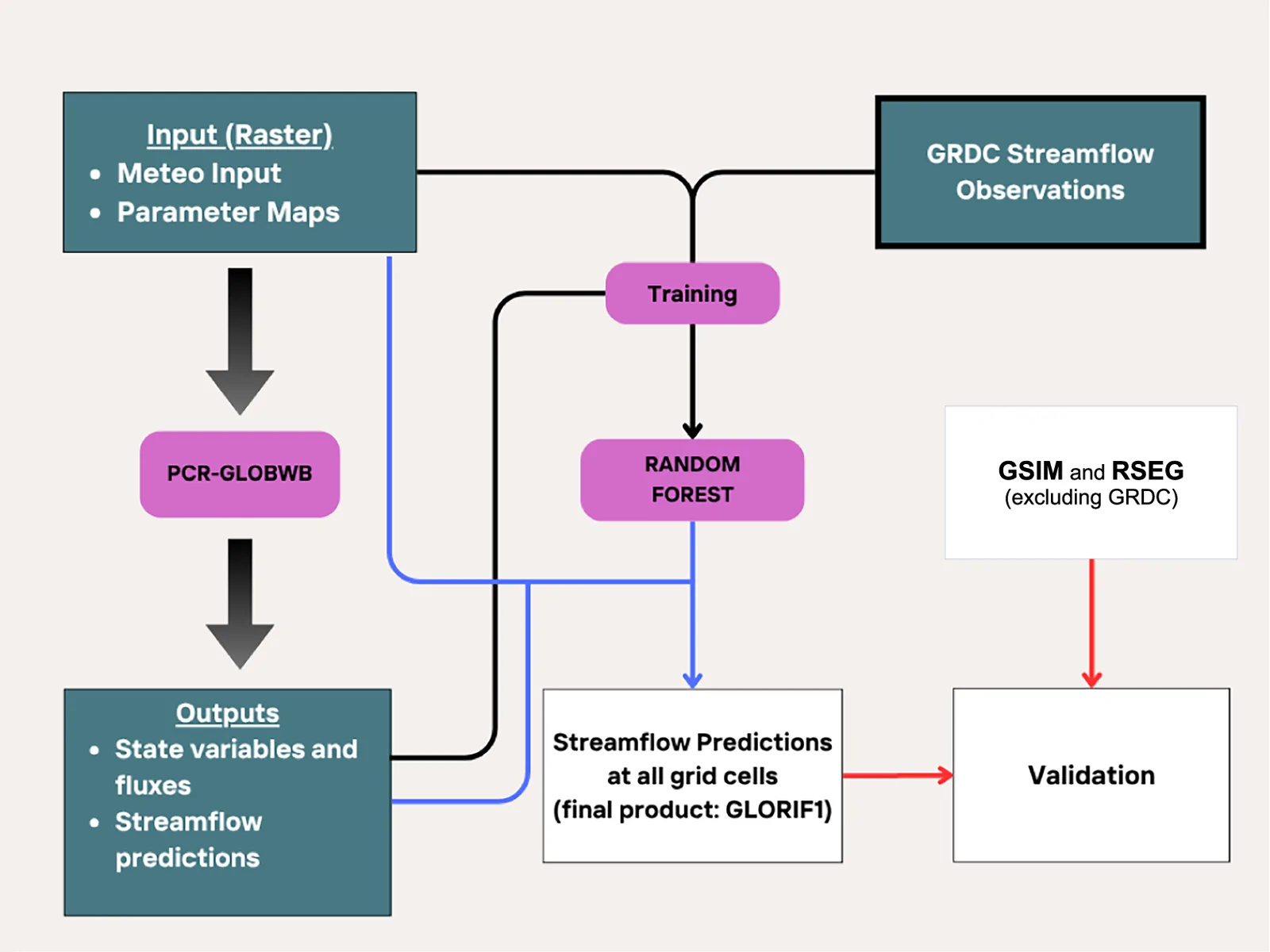
Schematic overview of the hybrid model that couples PCR-GLOBWB with Random Forest. Black lines illustrate the RF training process at GRDC station locations. Blue lines represent the application of RF for generating new streamflow predictions across all grid cells. Red lines highlight the validation process conducted to GSIM and RSEG datasets.

This study deploys the hybrid modeling approach that has been established in our previous study in Magni *et al*.^16^ to produce a comprehensive global gridded streamflow dataset. By using this method, the results are expected to be more accurate and realistic than those obtained directly from a standalone hydrological model. This dataset, named GLORIF1 (GLObal RIver Flow version 1)^17^, has its overall workflow illustrated in Fig. 1. The workflow begins with running the standalone PCR-GLOBWB hydrological model^7^ at a spatial resolution of 30 arcminutes (about 50 km at the equator). The PCR-GLOBWB model is driven by meteorological forcing variables and parameter inputs, generating state variables and streamflow predictions. These PCR-GLOBWB outputs, along with its inputs, are then used to train a RF algorithm using streamflow observation data from the GRDC. Once trained, the RF model generates new and improved streamflow estimates for 67,258 30-arcminute grid cells, providing global coverage. The performance of GLORIF1 is validated against approximately 2,000 independent observation stations from the GSIM. Moreover, comparison against RSEG (Remote Sensing-based Extension for the GRDC) is also done at approximately 1,000 stations.

## Methods

### PCR-GLOBWB

PCR-GLOBWB (PCRaster GLOBal Water Balance model)^7^ is a global hydrological model developed to simulate and analyze various components of the Earth’s water cycle at a global scale. For this work, the model was implemented at a resolution of 30 arcminutes and forced with the meteorological inputs from the W5E5 v2^18^ dataset. The model simulates streamflow and a wide range of hydrological state and flux variables, including the ones related to human water use and other anthropogenic activities, such as expansion of irrigated areas and construction of reservoirs^7,19^.

### Input data pre-processing for Random Forest

The variables from the PCR-GLOBWB are subsequently used in the RF algorithm. While PCR-GLOBWB runs at daily resolution, for the purpose of GLORIF1, we use its aggregated outputs at the monthly resolution, primarily to align with the temporal resolution of the more widely available GRDC observation data. Additionally, working with monthly data helps reduce computational demands.

Table S1 lists the input variables used in the Random Forest model as used in Magni *et al*.^16^, our previous study. These variables include meteorological inputs, flux and state variables, and catchment attributes. The catchment or upstream attribute data are primarily based on the PCR-GLOBWB model parameters. With the exception of the PCR-GLOBWB river discharge, labeled as pcrFlowDepth in Table S1, all other variables are processed through an upstream area procedure. Specifically, for each pixel and model parameter, the average value of all upstream pixels (from the given pixel to its most upstream location) is calculated and used as an input to the Random Forest model.

For the variable pcrFlowDepth, the original PCR-GLOBWB discharge values were converted into flow depth (m) using the following Equation (1)

1 $$D=\frac{Q}{A}\times 86400$$

where Q is the discharge (m^3^/s), A is the upstream area (m^2^) and D is the flow depth (m/day), the factor 86400 converts the discharge from a per-second rate to a daily rate.

All the variables in Table S1 were combined into predictor tables for each global 30 arc-min grid-cell. In total, 67,258 predictor tables were generated — corresponding to the number of 30-arcminute grid cells of PCR-GLOBWB — with each table stored as a separate CSV file.

To create the training dataset, a subset of 67,258 predictor tables was paired with corresponding discharge observations from the GRDC. The observation discharge values were also converted into flow depth using Equation (1), in line with the processing of PCR-GLOBWB discharge estimates, while their station locations were reassigned to the nearest corresponding PCR-GLOBWB cell centres. In principle we used the same GRDC data as used in Magni *et al*.^16^ (over 2000 stations). Nevertheless, to reduce the impact of outliers in the observation data used, such as those arising from spatial mismatches between GRDC station locations and the PCR-GLOBWB river networks, we applied a filtering procedure based on errors in average discharge values. Specifically, we calculated the 95th percentile of the absolute differences between the observed and PCR-GLOBWB average discharge. Stations with differences exceeding this threshold were considered outliers and excluded from the training dataset. This approach helped minimize the influence of potentially misaligned or inconsistent observations, ensuring a more reliable training set. Following this process, a total of 1,742 GRDC observation stations were ultimately used for training.

### Random Forest tuning & training

**Fig. 2:**
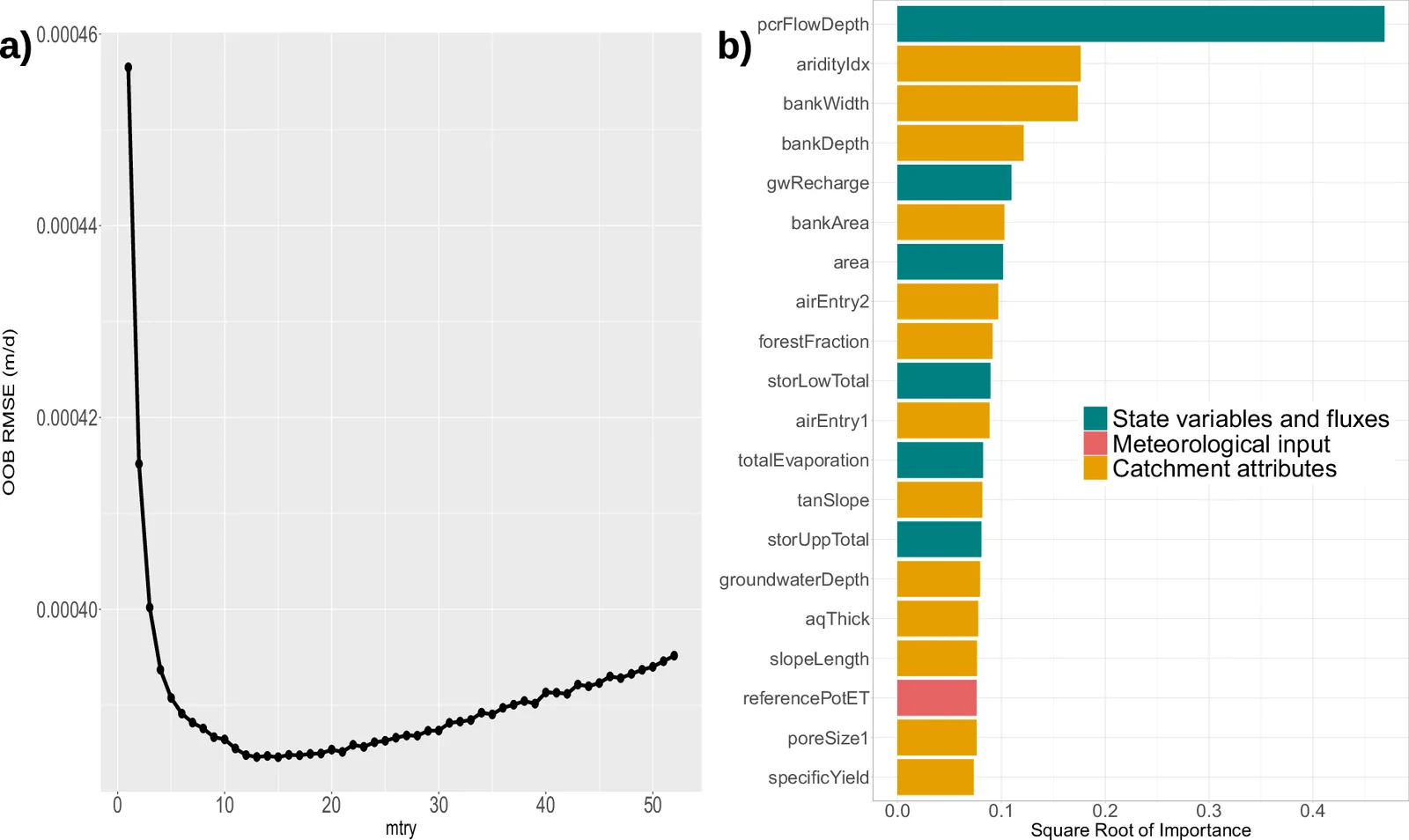
(**a**) Tuning of mtry, using a fixed ntree of 200. (**b**) Square-rooted mean decrease in impurity values of the top twenty variables. Each type of variable is represented by a different color. Explanation of codes in panel B is given in Table S1. mtry: the number of predictors randomly selected from the total number of available features at each split in the decision tree. OOB RMSE (Out-of-Bag Root Mean Squared Error): A metric that is calculated from the predictions made on the out-of-bag samples (data not used in training for a given tree) and measures the average magnitude of prediction errors. A lower OOB RMSE indicates better predictive accuracy.

RF model was implemented using the *ranger* package^20^ in R. To optimize the model, hyperparameter tuning focused on varying the *mtry* parameter, which determines the number of predictor variables randomly selected as candidates at each split. Specifically, the number of trees (*ntrees*) was set to 200, and *mtry* was tested from 1 up to the total number of predictors. The tuning function, *hyper_tuning*, trained a RF model for each value of *mtry*, evaluating each model using out-of-bag root mean squared error (OOB RMSE) as the performance metric. This process identified the *mtry* value that minimized OOB RMSE, thus improving the model’s generalization on unseen data. The tuning result, shown in Fig. 2a, revealed that the optimal *mtry* was 26. Once tuned, the final model was trained on the complete dataset, incorporating observational data alongside PCR-GLOBWB variables listed in Table S1.

The variable importance plotted in Fig. 2b shows the importance of each predictor variable. The importance of a feature in a RF model is determined by the decrease in impurity when the data is partitioned by that feature^21^. The square root of this decrease is used to interpret the relative importance of the features^22^. In this context, pcrFlowDepth stands out as the feature with the highest importance score, signifying its pivotal contribution to the model’s predictions. The following features in terms of importance, aridityIdx, bankWidth, and bankDepth, have significantly lower scores, emphasizing the dominant role of pcrFlowDepth.

The trained model was used to generate monthly flow depths for all 30-arcminute grid cells. These flow depth values were subsequently converted into discharge using the equation below.

2 $$Q=\frac{D\times A}{86400}$$

where Q is the discharge (m^3^/s), D is the flow depth (m/day), A is the upstream area (m^2^), and the division by 86400 adjusts the daily flow rate to a per-second rate.

For each cell, all predictions over time were saved in individual CSV files. These files were then consolidated into a single NetCDF file, organized by monthly time intervals, allowing for the analysis and visualization of the spatio-temporal distribution of discharge predictions across the globe.

### Quantile Random Forest (uncertainty quantification)

We also quantified the uncertainty of GLORIF1 using the Quantile Regression Forest (QRF) method based on Meinshausen^23^. We performed QRF and inferred the percentiles 2.5% and 97.5% of the predictive distribution of the corrected discharge time series. The QRF implemented used the same setup as the RF method used, including the same hyperparameter values and all predictors listed in Table S1.

### Validation to GSIM and RSEG

**Fig. 3:**
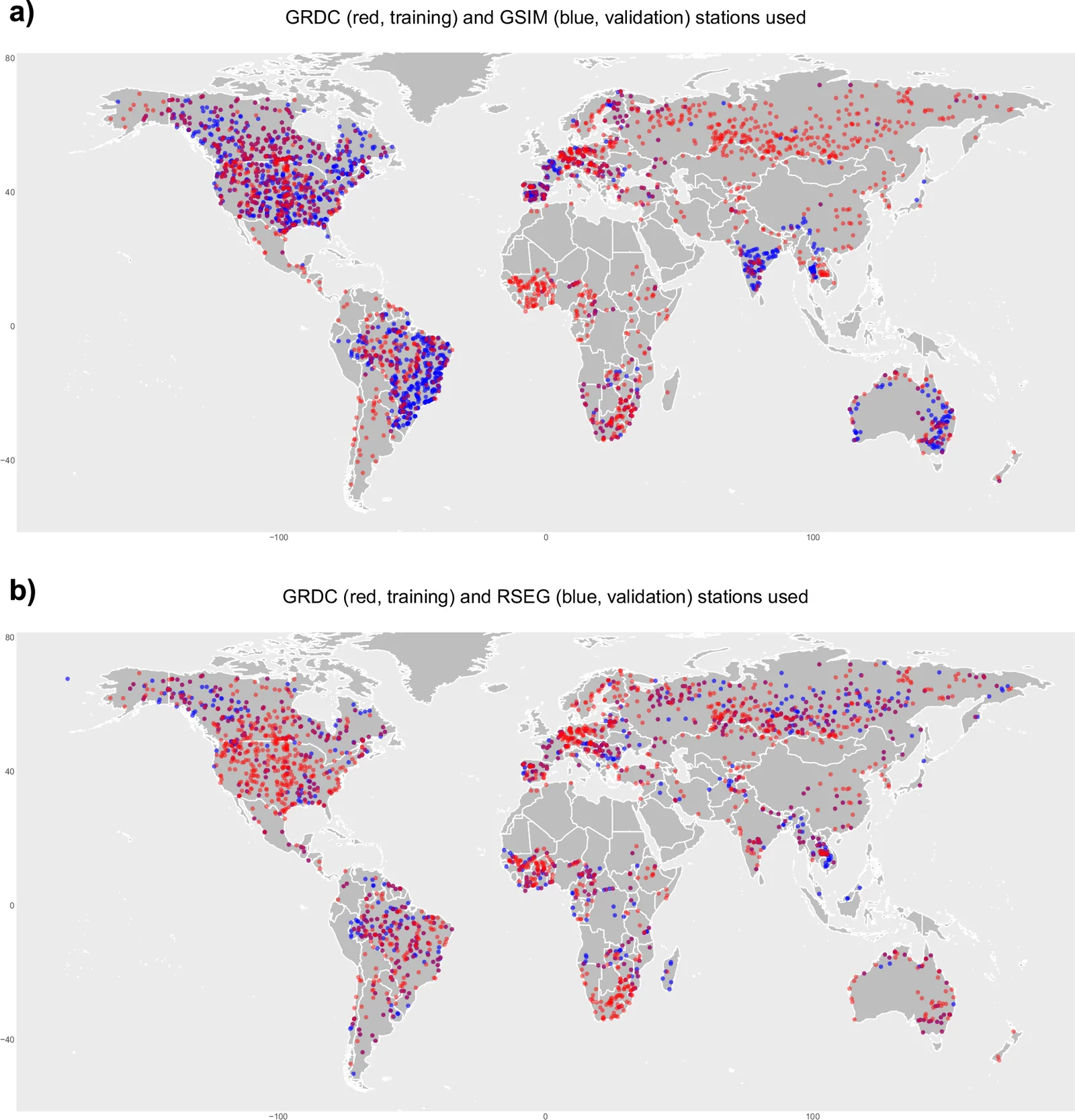
GRDC stations used for training (red) plotted above validation locations (blue) based on GSIM (**a**) and RSEG (**b**).

The validation data, sourced from GSIM^9,24^, generally underwent the same pre-processing as that applied to the GRDC data, including the alignment of station locations with the PCR-GLOBWB model output. GSIM contains a large number of discharge observation stations, each with varying data availability across different years. GSIM collects discharge data from multiple sources, such as national hydrological agencies, research institutions, global databases, data providers, and online platforms, including the GRDC data, which we used to train our RF model^9^. To prevent overlap with the GRDC data, stations with coordinates matching those of the GRDC stations used were filtered out. Additionally, due to the resolution limitations of the PCR-GLOBWB model used in this study, stations with catchment areas smaller than 10,000 km^2^ —roughly the size of four 30-arcminute grid cells — were excluded. Moreover, only stations with at least 12 monthly data points between 1979 and 2019 were selected. After applying these filtering criteria, the number of GSIM stations used for the validation was 1,986. Fig. 3a shows the spatial distribution of the GSIM stations used for this validation and GRDC stations used for the training. While in some regions their coverages are similar, notable differences still exist. These regions include Australia, Brazil, India, Thailand, and along China’s southern inland border with its neighbouring countries, where the GSIM stations cover areas that GRDC ones do not.

We also validated GLORIF1 to RSEG^25^, a remote sensing-based extension of GRDC river discharge dataset. RSEG uses satellite-derived observations of river width and water height to estimate monthly discharge and thereby extend GRDC time series. Because GRDC data were used to train our RF model, for the comparison with GLORIF1 we used only the remote sensing portions of the RSEG time series, removing any segments that belong to actual ground-based GRDC discharge observations. Also, we excluded stations with catchment areas smaller than 10,000 km^2^ and retained only stations with at least 12 monthly remote sensing-based data points. The number of RSEG stations used for this comparison was 996. Fig. 3b shows their spatial distribution.

## Data Records

**Fig. 4:**
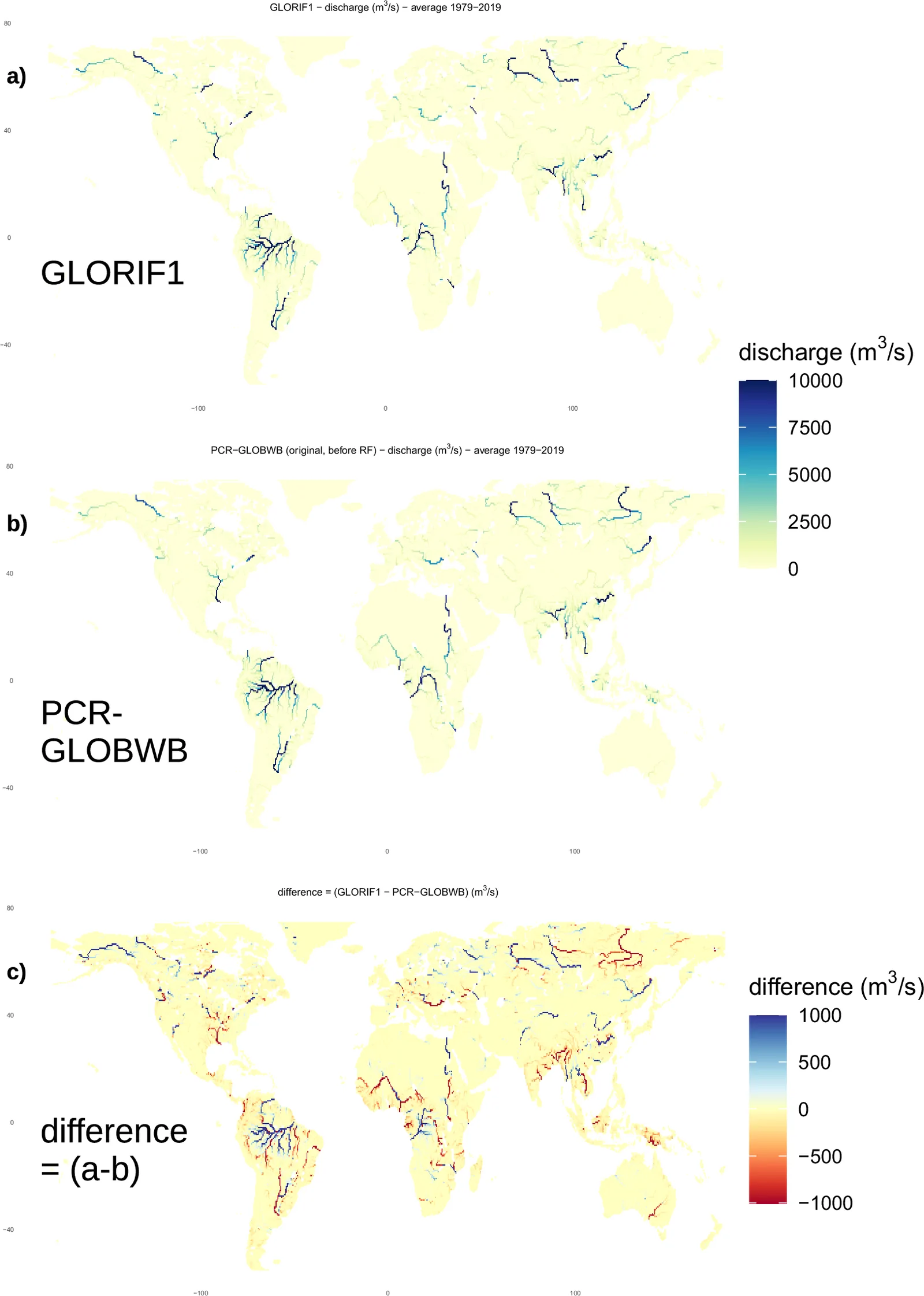
Discharge estimation maps for GLORIF1 and PCR-GLOBWB (m^3^/s). (**a**) Discharge values of GLORIF1, averaged over the years 1979 to 2019. (**b**) Discharge generated by the PCR-GLOBWB model, averaged over the years 1979 to 2019. (**c**) Difference between the average discharge values of GLORIF1 and PCR-GLOBWB, with blue color indicating GLORIF1 is higher than PCR-GLOBWB.

The data records associated with this work are stored on the Yoda data portal and can be found in the following location: https://doi.org/10.24416/UU01-1TZWLO^17^. The repository includes the original PCR-GLOBWB files used, as well as the observational GRDC and GSIM data, used for the RF training and validation. The resulting GLORIF1 discharge time series maps are stored in a NetCDF file (glorif1_discharge_30min_monthly.nc) at a resolution of 30 arcminutes and with monthly time steps. Additionally, the data has been averaged into a single NetCDF file using the cdo^26^ timmean function, which computes the mean over all input time steps on a pixel-by-pixel basis. For quick visualization, it is recommended to use ncview^27^, which provides an easy way to browse and visualize NetCDF files quickly. The averaged NetCDF file has also been converted into PCRaster^28^ maps using gdal_translate^29^ for easy visualization using Aguila^30^ software, which is part of the PCRaster^28^ software package. Figure 4 illustrates the results of the average discharge predictions and the differences between the original PCR-GLOBWB and GLORIF1 average discharge values. These visualizations provide a clear comparison between the original model outputs and the new predictions, highlighting areas of agreement and discrepancy. From the differences, we can observe whether some areas are getting wetter or drier. Compared to the original PCR-GLOBWB, GLORIF1 estimates higher discharge values in regions such as Amazon, while it predicts lower discharge in large parts of the African continent.

## Technical Validation

### Validation based on GSIM and RSEG

To assess the technical reliability of our dataset, we employed the Kling-Gupta Efficiency (KGE)^31^ metric, a quantitative approach for evaluating the performance of hydrological models by comparing simulated data to observed data. We validated GLORIF1 against the observed streamflow from 1,986 stations from the GSIM^9^ database.

**Fig. 5:**
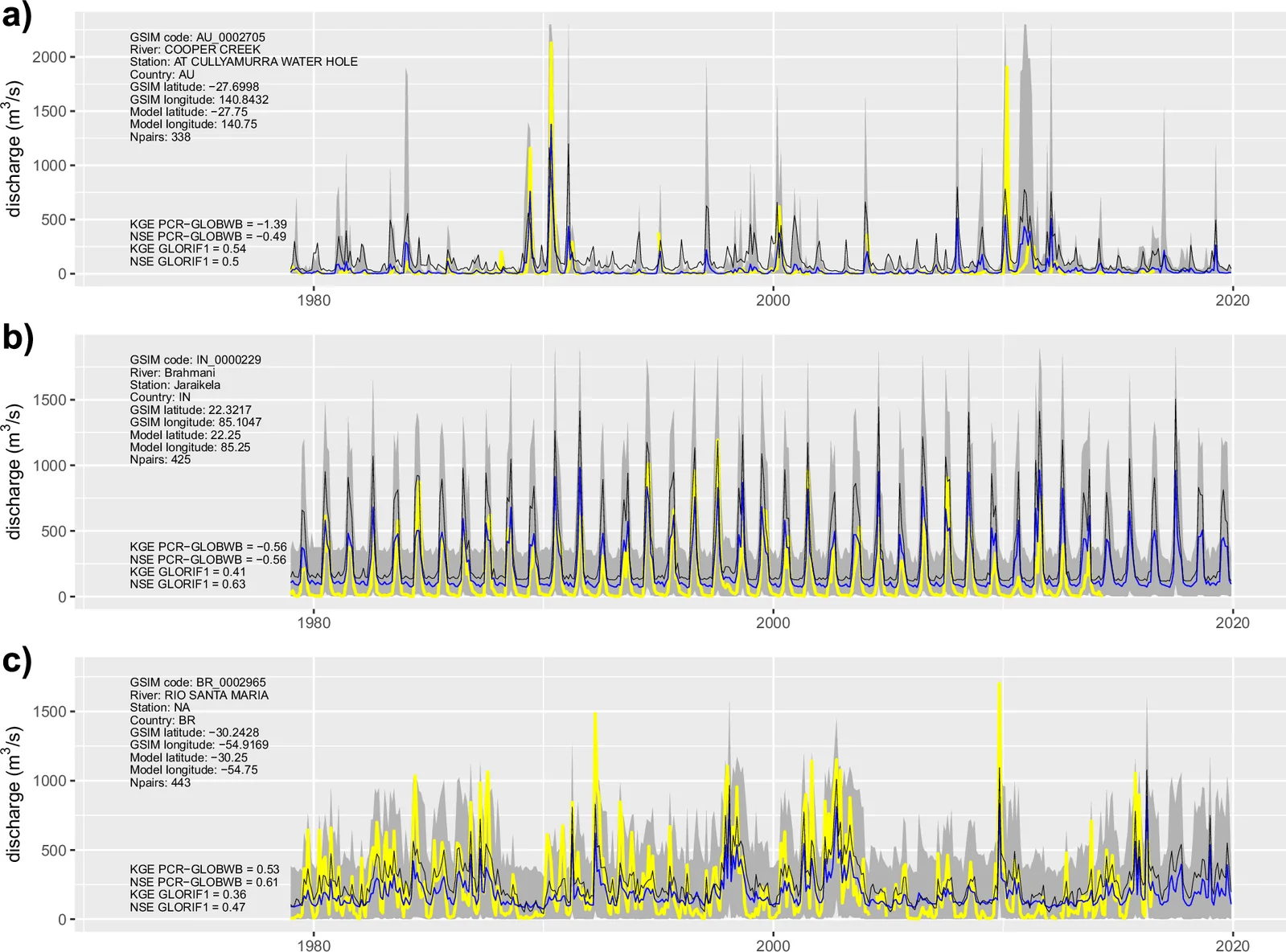
Discharge time series of PCR-GLOBWB (black, original), GLORIF1 (blue, after RF) and GSIM (yellow, observation) for three different locations. The shaded areas represent the range between percentiles 2.5% and 97.5% obtained from the QRF.

Figure 5a-c show, for three GSIM stations, the original discharge time series from the PCR-GLOBWB model and from the RF of GLORIF1, as well as those from the GSIM observations. The shaded areas represent the range between percentiles 2.5% and 97.5% of the corrected discharge time series obtained from the QRF. Figure 5a-b represent stations/locations with poor performance of the original PCR-GLOBWB, but after applying the RF model, performance improves as GLORIF1 time series are better aligned with observed discharge data. However, there are also some cases with decreased performance as illustrated in Fig. 5c. Nevertheless, overall, GLORIF outperforms PCR-GLOBWB. Fig. 7 illustrates the Kling-Gupta Efficiency (KGE) values for each individual station across the globe, comparing the performance from the original discharge time series from the PCR-GLOBWB model and those from GLORIF1. Three ranges of KGE values are distinguished. High KGE values above 0.6 indicate “Good” model performance. The values between  −0.41 and 0.6 are considered to be “Moderate”, while the ones below  −0.41 suggest “Poor” performance. As illustrated in Fig. 7, GLORIF1 demonstrates superior performance.

**Fig. 6:**
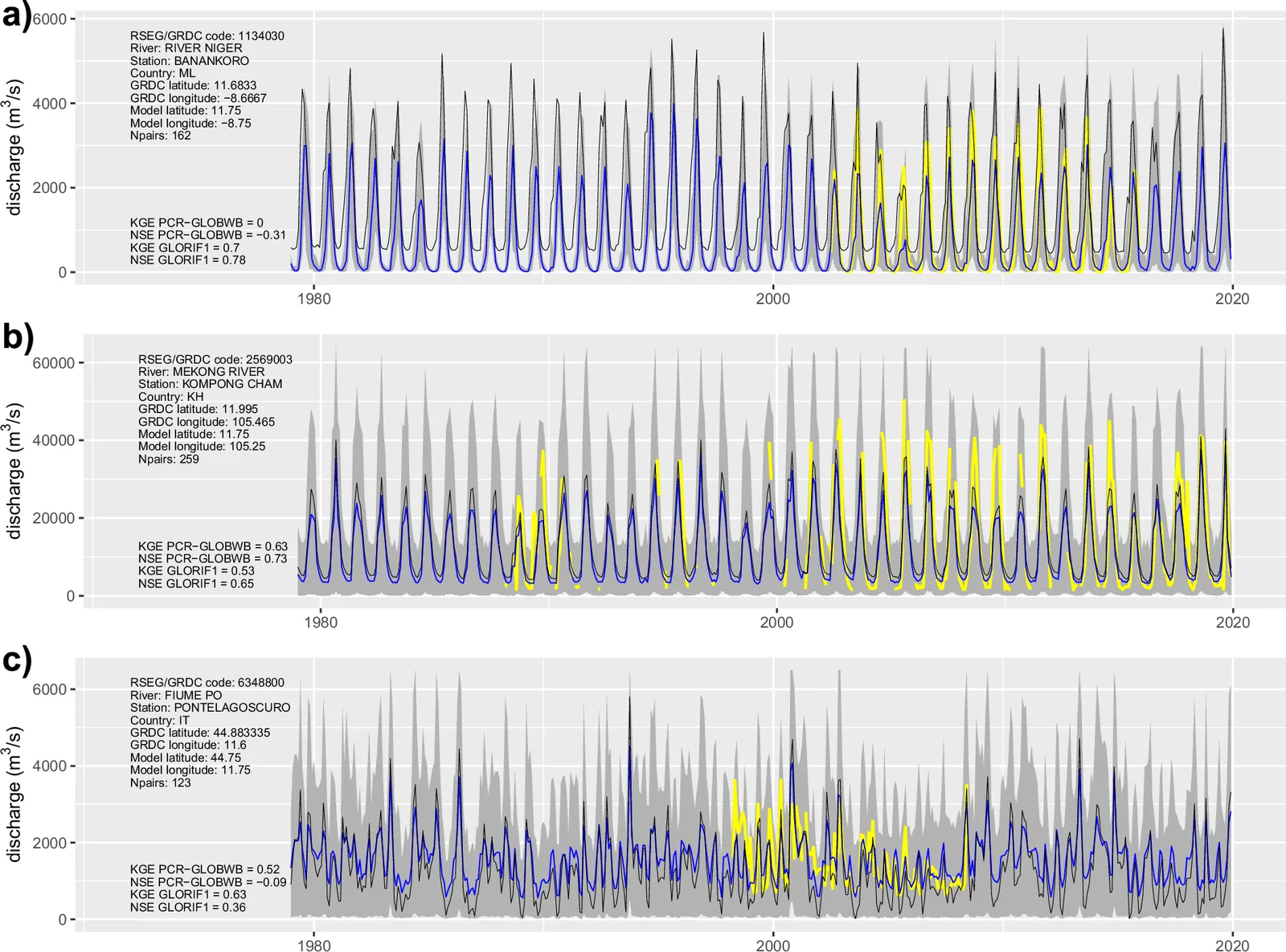
Discharge time series of PCR-GLOBWB (black, original), GLORIF1 (blue, after RF) and remote sensing-based RSEG (yellow) for three different locations. The shaded areas represent the range between percentiles 2.5% and 97.5% obtained from the QRF.

**Fig. 7:**
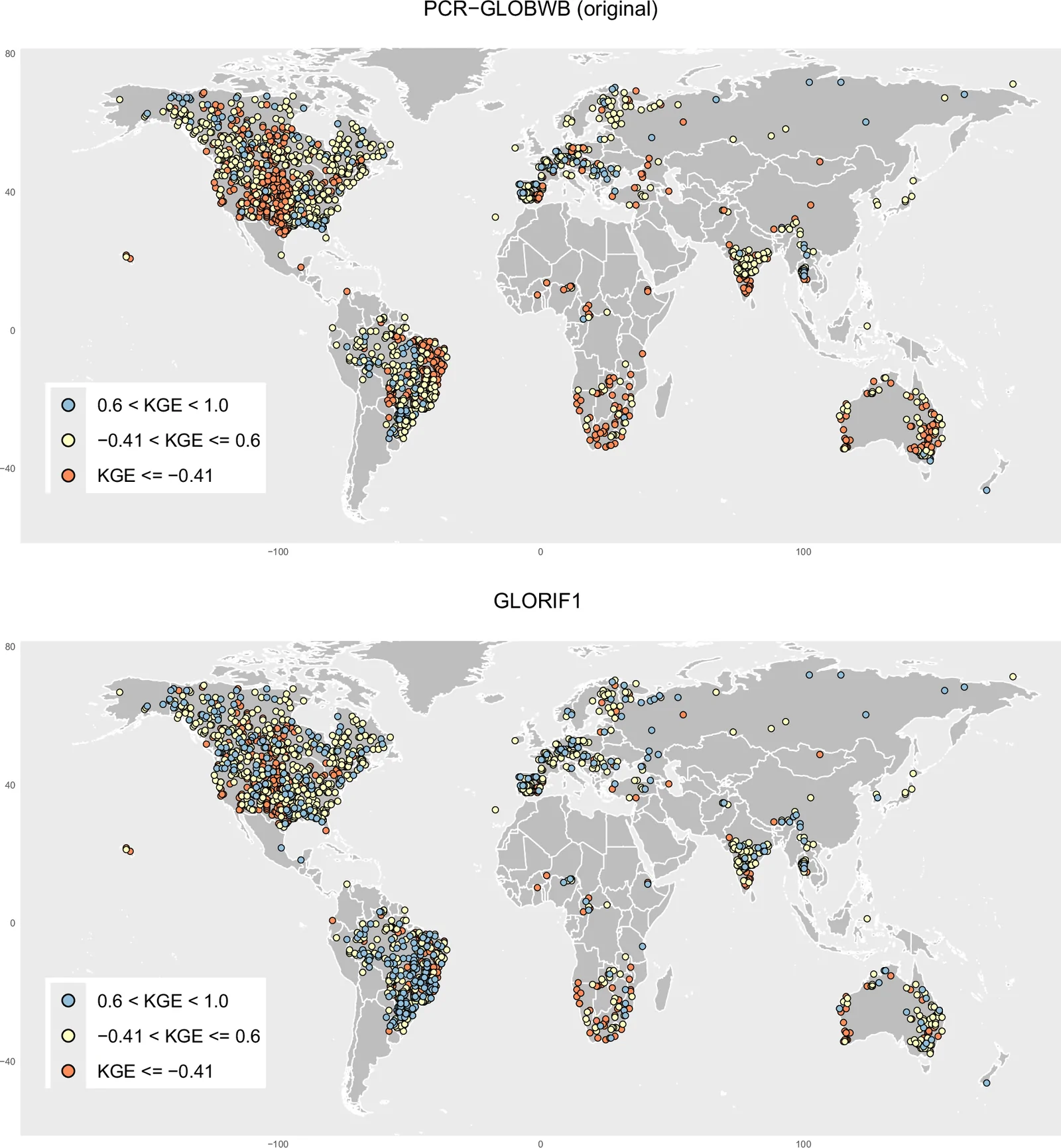
(**a**) KGE results of PCR-GLOBWB model for each station evaluated with GSIM. (**b**) KGE results of GLORIF1 for each station evaluated with GSIM. The color coding indicates different ranges of KGE values: Blue shades represent stations with high KGE values (0.6 < KGE < 1.0), indicating good model performance. Yellow shades denote moderate KGE values (−0.41 < KGE < = 0.6), suggesting satisfactory to mediocre model accuracy. Orange shades indicate poor performance (KGE  < = −0.41).

**Fig. 8:**
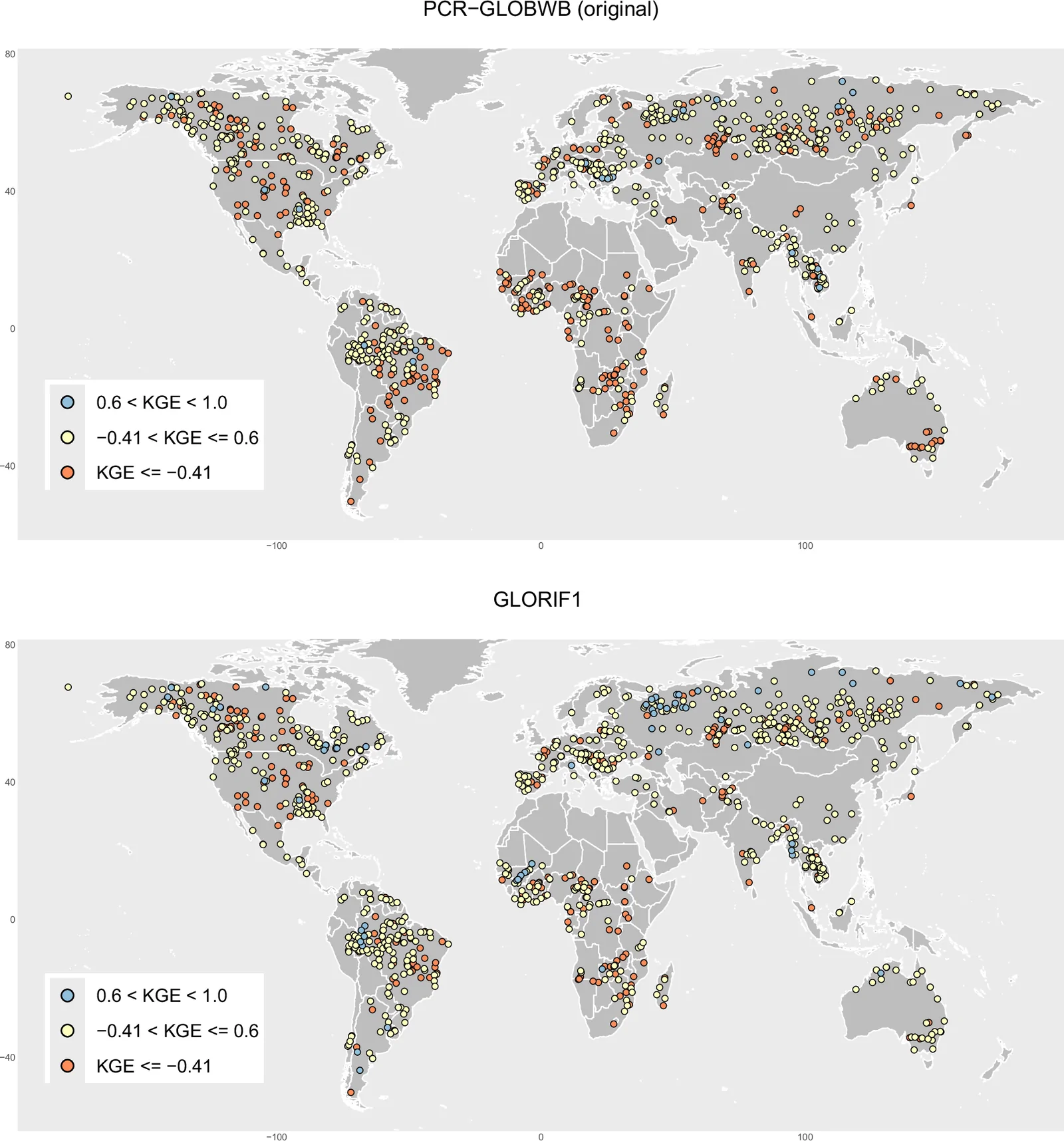
(**a**) KGE results of PCR-GLOBWB model for each station evaluated with RSEG. (**b**) KGE results of GLORIF1 for each station evaluated with RSEG. The color coding indicates different ranges of KGE values: Blue shades represent stations with high KGE values (0.6 < KGE < 1.0), indicating good model performance. Yellow shades denote moderate KGE values (−0.41 < KGE < = 0.6), suggesting satisfactory to mediocre model accuracy. Orange shades indicate poor performance (KGE < = −0.41).

**Fig. 9:**
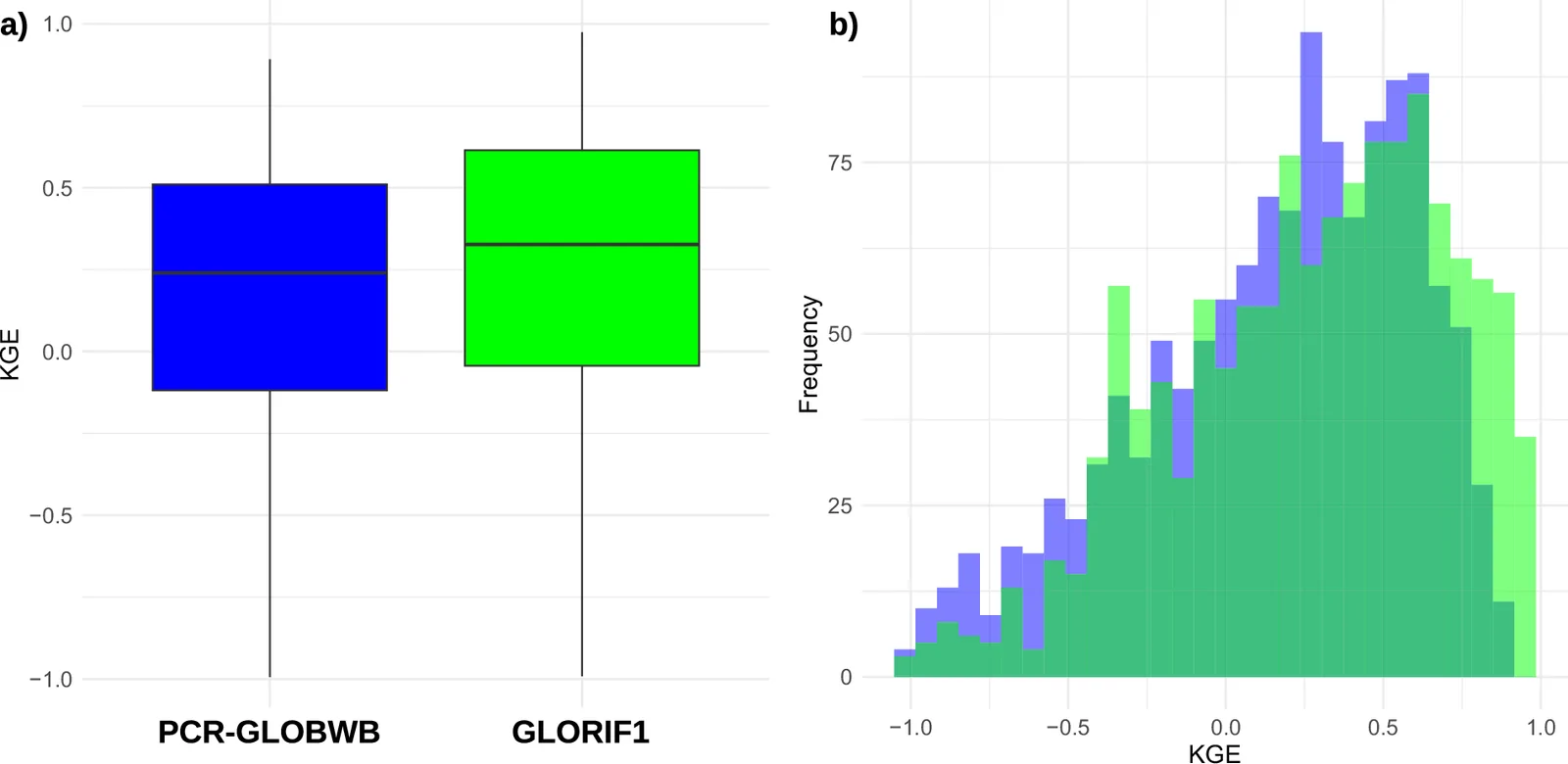
Evaluation to GSIM: (**a**) Boxplots of KGE values of PCR-GLOBWB and GLORIF1 predictions. (**b**) Histogram of KGE scores for PCR-GLOBWB and GLORIF1 predictions. Green stands for GLORIF1 and purple stands for PCR-GLOBWB.

Visual comparisons through box plots and histograms in Fig. 9 illustrate the performance differences between the GLORIF1 and PCR-GLOBWB. The box plots demonstrate that the GLORIF1 model consistently achieves higher Kling-Gupta Efficiency (KGE) scores, with a median score around 0.26, while the PCR-GLOBWB model has a median score close to 0.16. The distribution range for GLORIF1 is narrower, indicating less variability and more consistent performance across stations compared to the wider spread observed for PCR-GLOBWB. The histogram represents the distribution of KGE scores for two hydrological models, GLORIF1 and PCR-GLOBWB, across all stations. The GLORIF1 model demonstrates a more left-skewed distribution, with a peak around 0.75, indicating that most stations exhibit moderate to good performance under this model. The original PCR-GLOBWB model showcases a mixed performance, with fewer instances of good performance. The poor and moderate performances are nearly equally divided, suggesting that the model performs well at some stations but poorly at others. This pattern highlights the variability in PCR-GLOBWB’s performance, whereas GLORIF1 provides more consistent results.

The detailed analysis of KGE scores reveals that approximately 20% of stations in the GLORIF1 dataset fall into the “Good” category, compared to about 10% for the PCR-GLOBWB dataset. Both datasets have a significant proportion of stations classified as “Moderate”, with around 50% for GLORIF1 and 45% for PCR-GLOBWB. However, the PCR-GLOBWB model has a notably higher proportion of stations with “Poor” performance at 45%, while the GLORIF1 model has 30% in this category.

**Fig. 10:**
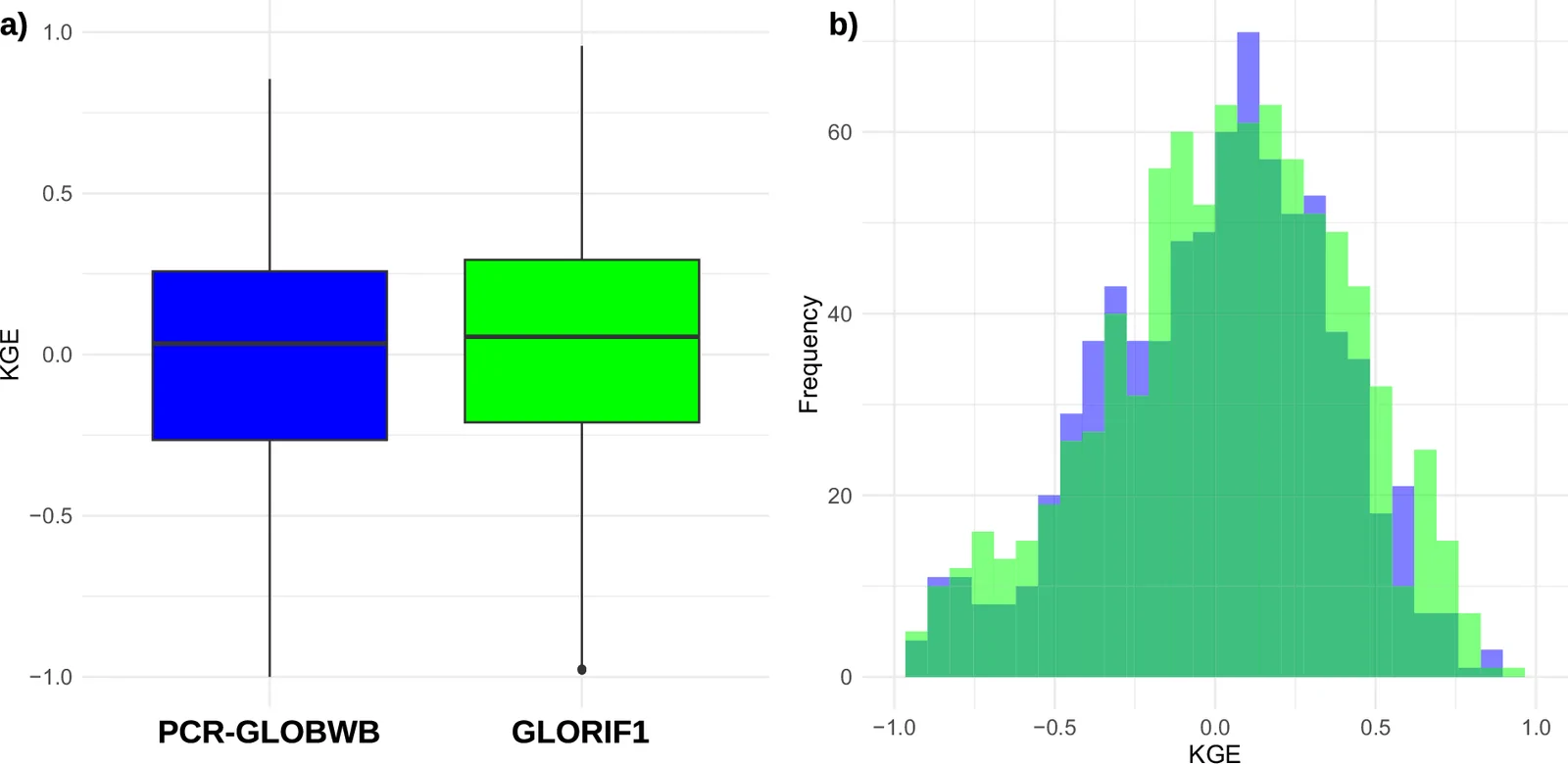
Evaluation to RSEG: (**a**) Boxplots of KGE values of PCR-GLOBWB and GLORIF1 predictions. (**b**) Histogram of KGE scores for PCR-GLOBWB and GLORIF1 predictions. Green stands for GLORIF1 and purple stands for PCR-GLOBWB.

Similarly to the aforementioned evaluation using GSIM, Fig. 6a-c plot, for three locations, the remote sensing-based RSEG time series together with their PCR-GLOBWB and GLORIF1 time series. Figure 6a exhibits an example of marked improvement with GLORIF1, while the others do not display comparable enhancements and in some cases show little to no improvement relative to the original PCR-GLOBWB time series. Figure 8 shows maps of KGE values for PCR-GLOBWB and GLORIF1 based on RSEG. Figure 10 shows their boxplots and historams. Overall, when benchmarked against RSEG, we observed a slight improvement in GLORIF1 compared with PCR-GLOBWB. However, this result should be interpreted with caution as the RSEG time series used are not actual ground-based data, but estimates derived from remote sensing.

### Validation based on global runoff

In addition to the Kling-Gupta Efficiency (KGE) evaluation, we validated GLORIF1 by calculating its global total runoff. This involved aggregating the runoff values at the outflow locations of streams to oceans or inland sinks and comparing them with established values reported in the literature (Table 1). The calculated global total runoff, averaged across all the years in our data, was 42,258 km^3^/year. This value closely matched the literature values of 41,867 km^3^/year (GRDC 2014, Report 44-WK14)^32^, 45,000 km^3^/year (Rodell *et al*., 2015)^33^, 42,811 km^3^/year (FAO, Aquastat 2016, online)^34^, and 42,393 km^3^/year (PCR-GLOBWB 2, 2018)^7^.

**Table 1:** Total global runoff values in this study and the literature (km^3^/year).

Source	Runoff (km^3^/year)
GLORIF1 (this study)	42,258
GRDC 2014, Report 44-WK14	41,867
Rodell *et al*. 2015	45,900 ± 4400
FAO, Aquastat 2016, online	42,811
PCR-GLOBWB 2, 2018	42,393

## Usage Notes

This dataset is particularly useful for global-scale hydrological analysis, offering valuable insights into streamflow patterns across large geographical areas. Nevertheless, it may not offer the accuracy required for small-scale applications, where local high resolution hydrological models or local observation data (if they are available) are more appropriate for precise estimate.

This release (https://public.yoda.uu.nl/geo/UU01/0OJ5HG.html) represents the first version of our dataset, featuring a spatial resolution of 30 arcminutes (about 50 km at the equator) and monthly temporal resolution. This dataset is offering valuable insights into streamflow spatial patterns and temporal dynamics across large geographical areas, and particularly useful for global-, continental, or large-scale hydrological analyses. Nevertheless, it may not offer the accuracy required for small-scale applications, where local high resolution hydrological models or local observation data (if they are available) are more appropriate for precise estimates. It is also not intended for short-term hydrological events at daily or hourly scales, such as flash floods. Future works will focus on enhancing spatial and temporal resolutions for subsequent versions.

## Supplementary information

Table S1: List of variables or features used in the Random Forest (RF) model

## Data Availability

The dataset is publicly available on the Yoda data portal of Utrecht University and can be found in the following location: https://doi.org/10.24416/UU01-1TZWLO. Please refer to the file “README.txt” for the content of the dataset.

## Code availability

Code is publicly available on GitHub and can be found on the following location: https://github.com/computationalgeography/GLORIF1.
