# Supplementary material for: GLORIF1, a global river flow dataset created by integrating process-based modelling and machine learning
Source: Sci Data. 2026 Jul 4;13:1482. doi: 10.1038/s41597-026-07658-6 (PMC13710845; doi:10.1038/s41597-026-07658-6)
Supplement: Supplementary file 1 [file 41597_2026_7658_MOESM1_ESM.pdf]

Supplementary Information

GLORIF1, a global river flow dataset created by integrating process-based modelling and machine learning

Sümeyye Büşra Işık[1,2,3], Oriol Pomarol Moya[1], Michele Magni[1], Madlene Nussbaum[1], Derek Karssenberg[1], and Edwin H. Sutanudjaja[1,*]

[1]Department of Physical Geography, Faculty of Geosciences, Utrecht University, Utrecht, The Netherlands
[2]Istanbul Technical University, Turkey
[3]State Hydraulic Works, Turkey
[*]corresponding author(s): Edwin H. Sutanudjaja (e.h.sutanudjaja@uu.nl)

This supplementary information contains Table S1: List of variables or features used in the Random Forest (RF) model.

Table S1. List of variables or features used in the Random Forest (RF) model, derived from the PCR-GLOBWB model. For further information of each of them, we refer to the References/sources column in the table.

Variable	Unit	Description	Data set	References/sources
pcrFlowDepth	m/day	Original PCR-GLOBWB flow depth	Flux Variable	PCR-GLOBWB[1]
precipitation	m/day	Meteo input – P	Meteorological Input	W5E5 v2[2]
temperature	°C	Meteo input – T	Meteorological Input	W5E5 v2[2]
referencePotET	m/day	Meteo input – PET	Meteorological Input	W5E5 v2[2]
baseflow	m/day	Groundwater discharge	Flux Variable	PCR-GLOBWB[1]
desalinationAbstraction	m/day	Water from desalination plants	Flux Variable	PCR-GLOBWB[1]
directRunoff	m/day	Surface runoff	Flux Variable	PCR-GLOBWB[1]
domesticWaterWithdrawal	m/day	Domestic water use	Flux Variable	PCR-GLOBWB[1]
fossilGroundwaterAbstractio	m/day	Non-renewable groundwater withdrawal	Flux Variable	PCR-GLOBWB[1]
gwRecharge	m/day	Groundwater recharge	Flux Variable	PCR-GLOBWB[1]
industryWaterWithdrawal	m/day	Industrial water use	Flux Variable	PCR-GLOBWB[1]
interflowTotal	m/day	Shallow sub-surface flow	Flux Variable	PCR-GLOBWB[1]
irrigationWaterWithdrawal	m/day	Irrigation water use	Flux Variable	PCR-GLOBWB[1]
livestockWaterWithdrawal	m/day	Livestock water use	Flux Variable	PCR-GLOBWB[1]
nonIrrWaterConsumption	m/day	Non-irrigation sectoral water consumption	Flux Variable	PCR-GLOBWB[1]
snowCoverSWE	m	Snow Water Equivalent	State Variable	PCR-GLOBWB[1]
snowFreeWater	m	Liquid water/meltwater storage in the snowpack	State Variable	PCR-GLOBWB[1]
storGroundwater	m	Groundwater storage (renewable)	State Variable	PCR-GLOBWB[1]
storLowTotal	m	S2 actual upper soil water storage	State Variable	PCR-GLOBWB[1]
storUppTotal	m	S1 actual lower soil water storage	State Variable	PCR-GLOBWB[1]
surfaceWaterAbstraction	m/day	Water withdrawal from surface water	Flux Variable	PCR-GLOBWB[1]
surfaceWaterInf	m/day	Surface water infiltration to groundwater	Flux Variable	PCR-GLOBWB[1]
surfaceWaterStorage	m	Surface water storage (lakes + reservoirs)	State Variable	PCR-GLOBWB[1]
totalEvaporation	m/day	Actual evapotranspiration	Flux Variable	PCR-GLOBWB[1]
totalGroundwaterAbstraction	m/day	Groundwater use (renewable + non-renewable)	Flux Variable	PCR-GLOBWB[1]
airEntry1	m	Air entry value (upper soil layer)	Catchment Attribute	FAO 2007[3], van Beek & Bierkens 2009[4]

Table S1. (continued)

Variable	Unit	Description	Data set	References/sources
airEntry2	m	Air entry value (lower soil layer)	Catchment Attribute	FAO 2007[3], van Beek & Bierkens 2009[4]
aqThick	m	Aquifer thickness	Catchment Attribute	GLHYMPS map, van Beek & Bierkens 2009[4]
area_pcr	m^2	Pixel upstream area	Catchment Attribute	HydroSHEDS[5], GTOPO30[6]
aridityIdx	-	Calculated as P/(P+PET) (1979-2019 mean)	Catchment Attribute	Calculated
bankArea	m^2	Calculated as bankDepth x bankWidth	Catchment Attribute	Calculated
bankDepth	m	Depth of riverbank	Catchment Attribute	HydroSHEDS[5], GTOPO30
bankWidth	m	Width of riverbank	Catchment Attribute	HydroSHEDS[5], GTOPO30
demAverage	m	Upstream elevation	Catchment Attribute	HydroSHEDS[5], GTOPO30
forestFraction	-	Upstream average forest fraction of total land use	Catchment Attribute	GLCC v2.0 map
groundwaterDepth	m	Pixel average groundwater depth (1979-2019)	Catchment Attribute	GLHYMPS map
KSat1	m/day	Saturated hydraulic conductivity (upper soil layer)	Catchment Attribute	FAO 2007[3], van Beek & Bierkens 2009[4]
KSat2	m/day	Saturated hydraulic conductivity (lower soil layer)	Catchment Attribute	FAO 2007[3], van Beek & Bierkens 2009[4]
kSatAquifer	m/day	Saturated hydraulic conductivity (aquifer)	Catchment Attribute	GLHYMPS[7] map
percolationImp	-	Fraction of area with impeded percolation	Catchment Attribute	van Beek 2008[8]
nporeSize1	-	Exponent in the soil water retention curve (upper soil layer)	Catchment Attribute	FAO 2007[3], van Beek & Bierkens 2009[4]
poreSize2	-	Exponent in the soil water retention curve (lower soil layer)	Catchment Attribute	FAO 2007[3], van Beek & Bierkens 2009[4]
recessionCoeff	day-1	Groundwater recession coefficient	Catchment Attribute	van Beek 2008[8]
resWC1	m3/m3	Residual water content (upper soil layer)	Catchment Attribute	FAO 2007[3], van Beek & Bierkens 2009[4]
resWC2	m3/m3	Residual water content (lower soil layer)	Catchment Attribute	FAO 2007[3], van Beek & Bierkens 2009[4]
satWC1	m3/m3	Saturated water content (upper soil layer)	Catchment Attribute	FAO 2007[3], van Beek & Bierkens 2009[4]
satWC2	m3/m3	Saturated water content (lower soil layer)	Catchment Attribute	FAO 2007[3], van Beek & Bierkens 2009[4]

Table S1. (continued)

**Variable**	**Unit**	**Description**	**Data set**	**References/sources**
slopeLength	m	Basin drainage density	Catchment Attribute	HydroSHEDS[5], GTOPO30
specificYield	m3/m3	Aquifer specific yield	Catchment Attribute	GLHYMPS map[7]
storage1	m	Water storage (upper soil layer)	State Variable	FAO 2007[3], van Beek & Bierkens 2009[4]
storage2	m	Water storage (lower soil layer)	State Variable	FAO 2007[3], van Beek & Bierkens 2009[4]
storDepth1	m	Depth (upper soil layer)	Catchment Attribute	FAO 2007[3]
storDepth2	m	Depth (lower soil layer)	Catchment Attribute	FAO 2007[3]
tanSlope	-	Pixel slope	Catchment Attribute	HydroSHEDS[5], GTOPO30[6]

References

1. Sutanudjaja, E. H. *et al.* PCR-GLOBWB 2: a 5 arcmin global hydrological and water resources model. *Geosci. Model. Dev.* **11**, 2429–2453, 10.5194/gmd-11-2429-2018 (2018).
2. Lange, S. *et al.* WFDE5 over land merged with ERA5 over the ocean (W5E5 v2.0), 10.48364/ISIMIP.342217 (2021).
3. Food and Agriculture Organization of the United Nations (FAO). Digital soil map of the world, version 3.6 (2007).
4. van Beek, L. P. H. & Bierkens, M. F. P. The Global Hydrological Model PCR-GLOBWB: Conceptualization, Parameterization and Verification. Tech. Rep., Department of Physical Geography, Utrecht University, Utrecht, the Netherlands, https://vanbeek.geo.uu.nl/suppinfo/vanbeekbierkens2009.pdf (2009). Last accessed: 2 August 2024.
5. Lehner, B., Verdin, K. & Jarvis, A. New Global Hydrography Derived From Spaceborne Elevation Data. *Eos, Transactions Am. Geophys. Union* **89**, 93–94, 10.1029/2008eo100001 (2008).
6. Earth Resources Observation And Science (EROS) Center. Global 30 Arc-Second Elevation (GTOPO30), 10.5066/F7DF6PQS (2017).
7. Gleeson, T., Moosdorf, N., Hartmann, J. & van Beek, L. P. H. A glimpse beneath earth's surface: GLobal HYdrogeology MaPS (GLHYMPS) of permeability and porosity. *Geophys. Res. Lett.* **41**, 3891–3898, 10.1002/2014gl059856 (2014).
8. van Beek, L. P. H. Forcing PCR-GLOBWB with CRU data. Tech. Rep., Department of Physical Geography, Utrecht University, Utrecht, the Netherlands, https://vanbeek.geo.uu.nl/suppinfo/vanbeek2008.pdf (2008). Last accessed: 5 August 2024.
